# The added value of user involvement during the development of a feedback system regarding physical functioning for community-dwelling elderly people

**Published:** 2012-06-15

**Authors:** Joan Vermeulen, Jacques C. L Neyens, Marieke D Spreeuwenberg, Erik Van Rossum, David Hewson, Luc P De Witte

**Affiliations:** Maastricht University, Maastricht, The Netherlands; Maastricht University, Maastricht, The Netherlands; Maastricht University, Maastricht; Zuyd University of Applied Sciences, Heerlen, The Netherlands; Maastricht University, Maastricht; Zuyd University of Applied Sciences, Heerlen, The Netherlands; Université de Technologie de Troyes, Troyes, France; Maastricht University, Maastricht, Zuyd University of Applied Sciences, Heerlen, The Netherlands

**Keywords:** user-centered design, elderly people, self management, physical functioning, usability

## Abstract

**Background:**

The number of frail elderly people is increasing. Unfortunately, the number of caregivers is not increasing at the same pace, which affects older people, caregivers and healthcare systems. Because of these developments, self-management is becoming more important in healthcare. To support community-dwelling elderly people in their self-management, a system was developed that monitors their physical functioning. This system provides feedback to elderly people and their caregivers regarding physical indicators of frailty. The feedback is provided to elderly people via the screen of a mobile phone. It is important that elderly people understand the content of the feedback and are able to use the mobile phone properly. If not, it is unlikely that the system can support self-management. Many interactive health technologies that have been developed do not fulfil their promises. An important reason for this is that human and other non-technology issues are not sufficiently taken into consideration during the development process.

**Objective:**

To collaborate with elderly people during the development and evaluation of a feedback system for community-dwelling elderly people regarding physical functioning.

**Methods:**

An iterative user-centered design that consists of five phases was used to develop and evaluate the feedback system. These five phases were: 1) Selection of users, 2) Analysis of users and their context, 3) Identification of user needs, 4) Development of a prototype, and 5) Evaluation of the prototype. Three representatives of a target group panel for elderly people were selected in phase 1. They shared their needs and preferences during three expert group meetings that took place during the development process. This resulted in the development of a prototype which was first evaluated in a heuristic evaluation. Once adjustments were made, 11 elderly people evaluated the adjusted prototype using a think aloud procedure. They rated the usability and acceptability of the developed interface on a scale from 1 till 7 using an adapted version of the Post-Study System Usability Questionnaire (PSSUQ).

**Results:**

A feedback system was developed that provides feedback regarding physical indicators of frailty via a touch screen mobile phone. The interface uses colours, smiley’s, and spoken/written messages to provide feedback that is easy to understand. The heuristic evaluation revealed that there were some problems with consistency and the use of user language. The think aloud evaluation showed that the 11 elderly people were able to navigate through the interface without much difficulty despite some small problems related to the lay-out of the interface. The mean score on an adapted version of the PSSUQ was 5.90 (SD 1.09) which indicates high user satisfaction and good usability.

**Conclusions:**

The involvement of end-users significantly influenced the lay-out of the interface that was developed. This resulted in an interface that was accepted by the target group. Evaluation of the prototype revealed that the usability of the interface was good. The feedback system will only succeed in supporting self-management when elderly people are able to use the interface and understand the feedback. The input of elderly people during the development process contributed to this.

